# Succinimide Formation from an NGR-Containing Cyclic Peptide: Computational Evidence for Catalytic Roles of Phosphate Buffer and the Arginine Side Chain

**DOI:** 10.3390/ijms18020429

**Published:** 2017-02-16

**Authors:** Ryota Kirikoshi, Noriyoshi Manabe, Ohgi Takahashi

**Affiliations:** Faculty of Pharmaceutical Sciences, Tohoku Medical and Pharmaceutical University, 4-4-1 Komatsushima, Aoba-ku, Sendai 981-8558, Japan; kirikoshi@tokoku-mpu.ac.jp (R.K.); manabe@tohoku-mpu.ac.jp (N.M.)

**Keywords:** Asn-Gly-Arg (NGR) motif, *iso*Asp-Gly-Arg (*iso*DGR) motif, tumor-targeting ligands, deamidation, nonenzymatic reaction, succinimide intermediate, phosphate buffer catalysis, intramolecular catalysis, computational chemistry, density functional theory

## Abstract

The Asn-Gly-Arg (NGR) motif and its deamidation product *iso*Asp-Gly-Arg (*iso*DGR) have recently attracted considerable attention as tumor-targeting ligands. Because an NGR-containing peptide and the corresponding *iso*DGR-containing peptide target different receptors, the spontaneous NGR deamidation can be used in dual targeting strategies. It is well known that the Asn deamidation proceeds via a succinimide derivative. In the present study, we computationally investigated the mechanism of succinimide formation from a cyclic peptide, c[CH_2_CO-NGRC]-NH_2_, which has recently been shown to undergo rapid deamidation in a phosphate buffer. An H_2_PO_4_^−^ ion was explicitly included in the calculations. We employed the density functional theory using the B3LYP functional. While geometry optimizations were performed in the gas phase, hydration Gibbs energies were calculated by the SM8 (solvation model 8) continuum model. We have found a pathway leading to the five-membered ring tetrahedral intermediate in which both the H_2_PO_4_^−^ ion and the Arg side chain act as catalyst. This intermediate, once protonated at the NH_2_ group on the five-membered ring, was shown to easily undergo NH_3_ elimination leading to the succinimide formation. This study is the first to propose a possible catalytic role for the Arg side chain in the NGR deamidation.

## 1. Introduction

**Scheme 1:**
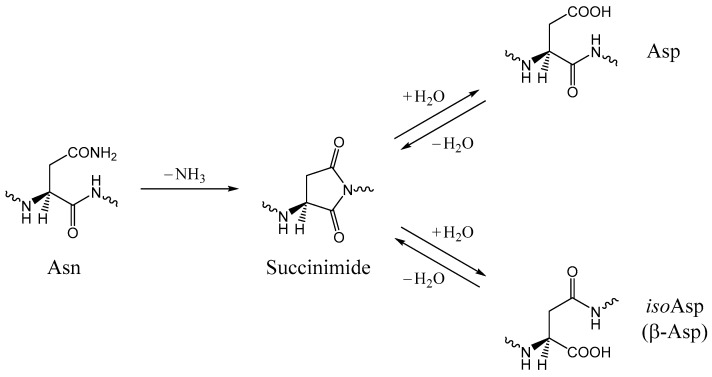
Succinimide-mediated deamidation of an Asn residue giving *iso*Asp (β-Asp) and Asp residues.

**Scheme 2:**
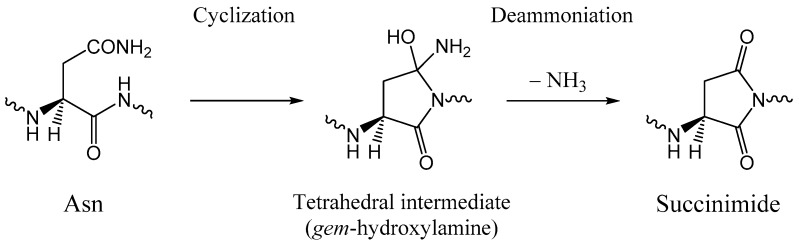
Two-step (cyclization-deammoniation) mechanism for the succinimide formation from an Asn residue.

Deamidation of asparagine (Asn, N) residues is one of the most common reactions which occur nonenzymatically in peptide chains. A succinimide species is known to be the intermediate of Asn deamidation (Scheme 1) [1,2,3,4,5,6,7,8,9,10,11]. This intermediate having a five-membered ring is formed by the nucleophilic attack of the main-chain nitrogen atom of the C-terminal adjacent residue on the Asn side-chain amide carbon with release of an ammonia molecule. This is an intramolecular nucleophilic substitution reaction and is generally considered to occur in two steps (cyclization-deammoniation, Scheme 2) [12,13]. In the first step, the nucleophilic attack gives rise to a five-membered ring tetrahedral intermediate. In the second step, an NH_3_ molecule is released from this intermediate to give the succinimide species. Hydrolysis of the succinimide intermediate can occur at either of its two carbonyl groups, leading to the formation of aspartic acid (Asp, D) and isoaspartic acid (β-aspartic acid) (*iso*Asp, *iso*D) residues in a typical ratio of 1:3 [1,2,3,4,5,6,7]. Small amounts of d-Asp and d-*iso*Asp residues may also be formed via racemization of the succinimide intermediate [1,7,14,15]. While the Asn deamidation in proteins and peptides is often regarded as a degradation reaction, a “molecular clock” hypothesis was proposed which claims that the Asn deamidation regulates the timing of biological processes such as protein turnover [8,9,10]. It is well known that the rates of Asn deamidation are greatly dependent on the neighboring amino acid residue on the C-terminal side and are generally by far the fastest when this residue is glycine (Gly, G), the smallest amino acid residue [1,3,5,6,7,8,9].

Recently, peptides containing the asparagine-glycine-arginine (Asn-Gly-Arg, NGR) motif [16,17,18,19,20,21,22,23,24,25,26,27,28,29,30,31,32,33,34,35,36,37,38,39,40,41,42,43,44,45,46,47,48,49,50,51,52,53,54,55,56,57,58,59,60,61,62,63,64,65,66,67,68,69,70,71,72,73,74,75] and their deamidation products containing the *iso*DGR motif [38,41,46,48,56,59,74,76,77,78,79,80,81,82,83,84,85] have attracted considerable attention as tumor-targeting ligands. The tumor-targeting property of the NGR motif was first reported by Ruoslahti and co-workers in 1998 [16], and then it was revealed that peptides containing the NGR motif bind to an aminopeptidase N (APN or CD13) isoform which is uniquely expressed on the endothelium of tumor vasculature [18,22]. It was later that the *iso*DGR-containing peptides (but not DGR-containing peptides), especially cyclic ones, bind to α_v_β_3_ integrin which is also overexpressed in tumor cells including the vascular endothelium [38,48,76,77]. NGR-containing peptides thus may be used for dual targeting strategies in specifically targeted delivery of various drugs, imaging agents, etc., to tumors [38,41,59,71,74,82], and clarifying the detailed mechanism of the NGR-to-*iso*DGR transition will be helpful in designing dual targeting ligands with desired properties.

**Figure 1:**
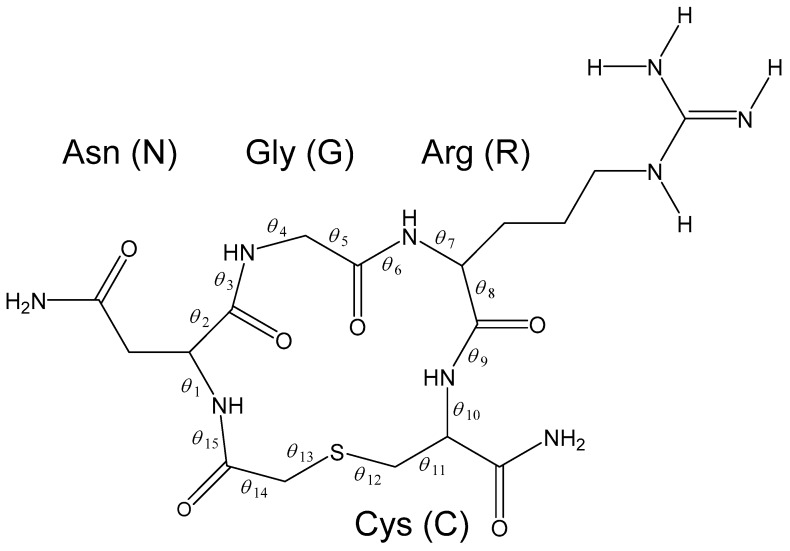
A 15-membered cyclic peptide, c[CH_2_CO-NGRC]-NH_2_, containing the NGR (Asn-Gly-Arg) motif. This peptide is called CP15 in this paper. The guanidino group of the Arg side chain is depicted as a deprotonated form (see text for details). The definition of the heavy-atom dihedral angles *θ*_1_–*θ*_15_ for the 15-membered ring are also shown.

Very recently, Enyedi and co-workers have shown that several NGR-containing cyclic peptides with a 15- to 18-membered ring undergo very rapid deamidation [71]. Among those, c[CH_2_CO-NGRC]-NH_2_ (Figure 1) having a cysteine (Cys, C) thioether linkage in a 15-membered ring deamidated most rapidly in phosphate-buffered saline (PBS, pH 7.4) at room temperature. More specifically, 68% and 17% of the peptide were converted to the corresponding *iso*DGR and DGR peptides, respectively, after 48 h incubation. It should be noted that the peptide was stable in distilled or slightly acidic water at room temperature for 48 h. Therefore, the phosphate buffer is considered to catalyze the deamidation. Hereafter, we denote the 15-membered cyclic peptide, c[CH_2_CO-NGRC]-NH_2_, as CP15.

In a recent paper, we have computationally shown that glycolic acid (in its protonated form) can catalyze Asn deamidation at a low pH [12]. In the mechanism we have revealed, a glycolic acid molecule acts as both proton donor and acceptor in double proton transfers. A similar mechanism may also operate in the phosphate-catalyzed deamidation in the NGR motif.

In the present study, we have computationally revealed a phosphate-catalyzed mechanism for the succinimide formation from CP15. The Arg residue also plays a catalytic role in that it contributes to fixing a catalytic H_2_PO_4_^−^ ion in the right position for the first step (i.e., the formation of the tetrahedral intermediate). It is also shown that the tetrahedral intermediate, once protonated at the NH_2_ group on the five-membered ring, easily releases an NH_3_ molecule to form the succinimide species.

## 2. Results and Discussion

**Figure 2:**
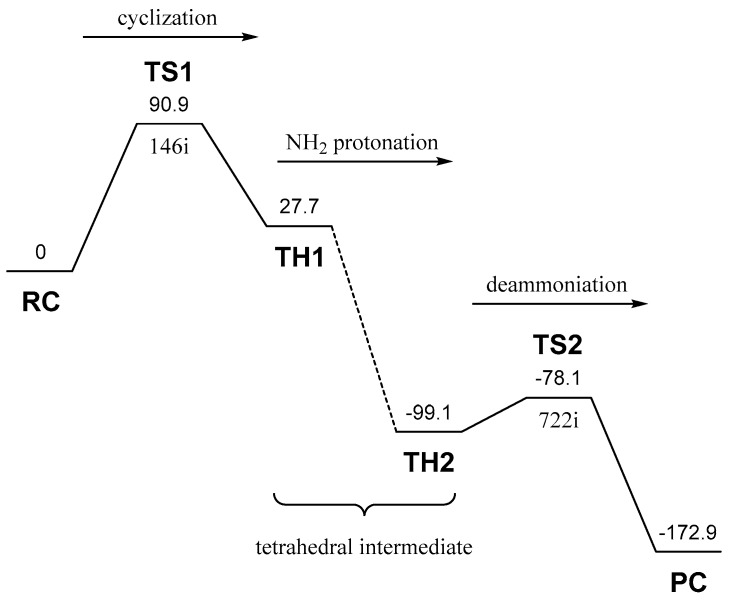
Energy profile in water. RC: reactant complex; TS: transition state; TH: tetrahedral intermediate; PC: product complex. The relative energies with respect to RC are shown in kJ·mol^−1^. The imaginary frequencies for the two transition states are also shown.

**Figure 3:**
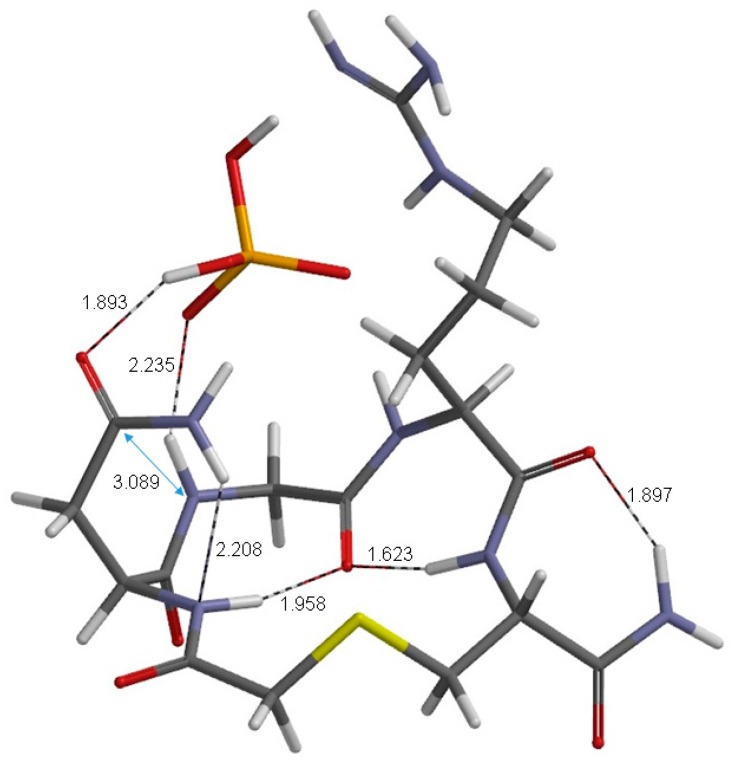
The geometry of the reactant complex (RC) formed between the cyclic peptide CP15 (in a deprotonated form as shown in Figure 1) and an H_2_PO_4_^−^ ion. Selected interatomic distances are shown in Å. Grey: carbon; white: hydrogen; blue: nitrogen; red: oxygen; orange: phosphorus; yellow: sulfur.

**Figure 4:**
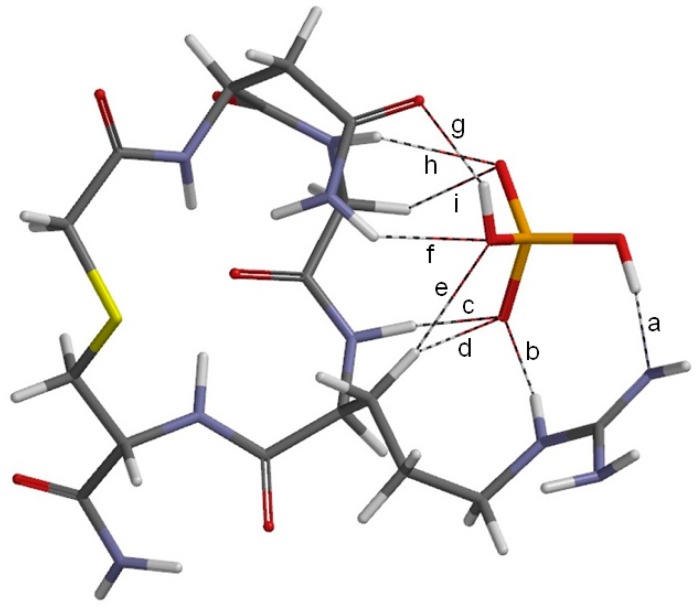
The nine-point interaction (interactions a–i) in RC (the reactant complex shown in Figure 3). The corresponding interatomic distances are shown in Table 1. Grey: carbon; white: hydrogen; blue: nitrogen; red: oxygen; orange: phosphorus; yellow: sulfur.

**Figure 5:**
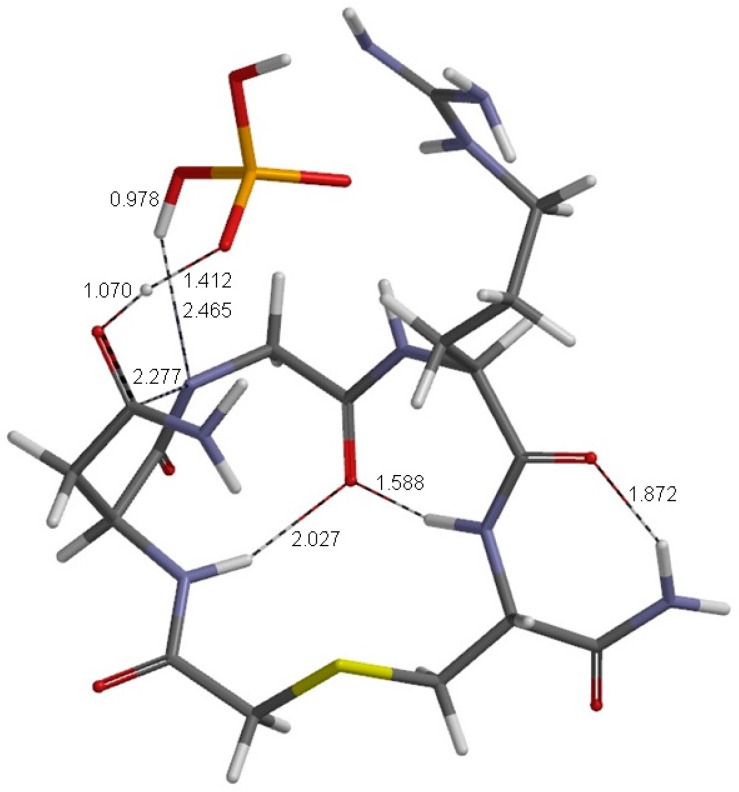
The geometry of TS1 (transition state of the first step) connecting RC (Figure 3) and TH1 (tetrahedral intermediate directly connected to TS1) (Figure 6). Selected interatomic distances are shown in Å. Grey: carbon; white: hydrogen; blue: nitrogen; red: oxygen; orange: phosphorus; yellow: sulfur.

**Figure 6:**
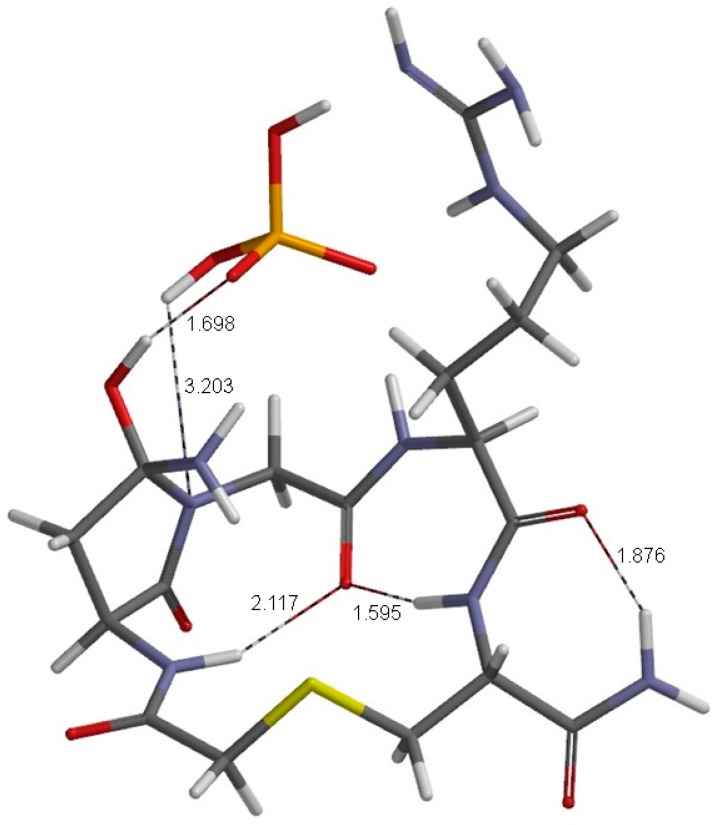
The geometry of TH1, the tetrahedral intermediate directly connected to TS1 (Figure 5). Selected interatomic distances are shown in Å. Grey: carbon; white: hydrogen; blue: nitrogen; red: oxygen; orange: phosphorus; yellow: sulfur.

**Figure 7:**
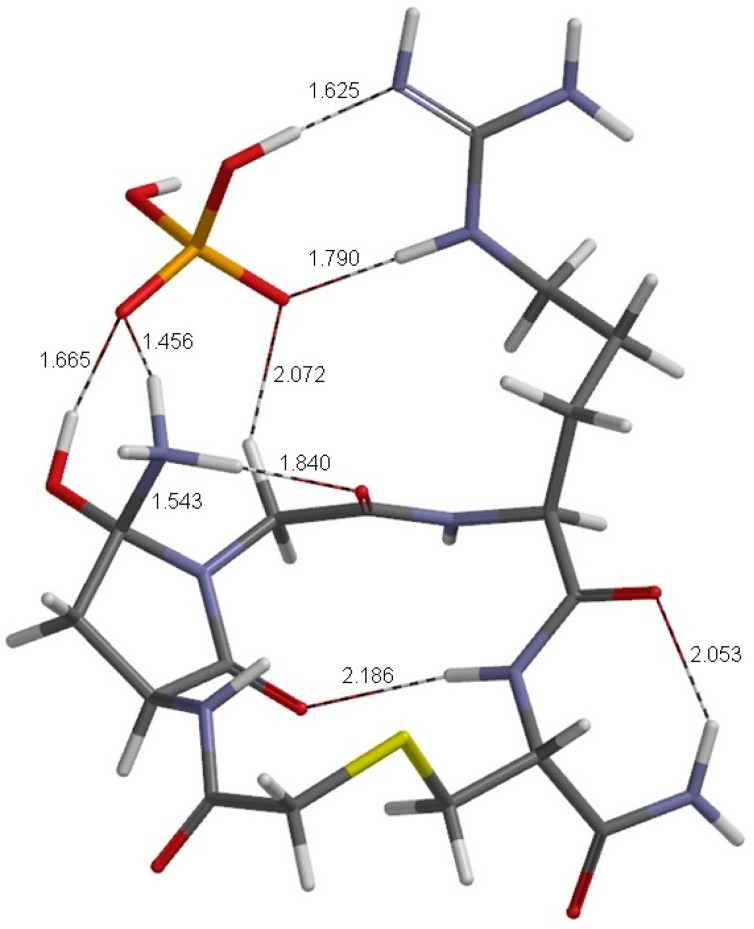
The geometry of TH2, the protonated tetrahedral intermediate directly connected to TS2 (Figure 8). Selected interatomic distances are shown in Å. Grey: carbon; white: hydrogen; blue: nitrogen; red: oxygen; orange: phosphorus; yellow: sulfur.

**Figure 8:**
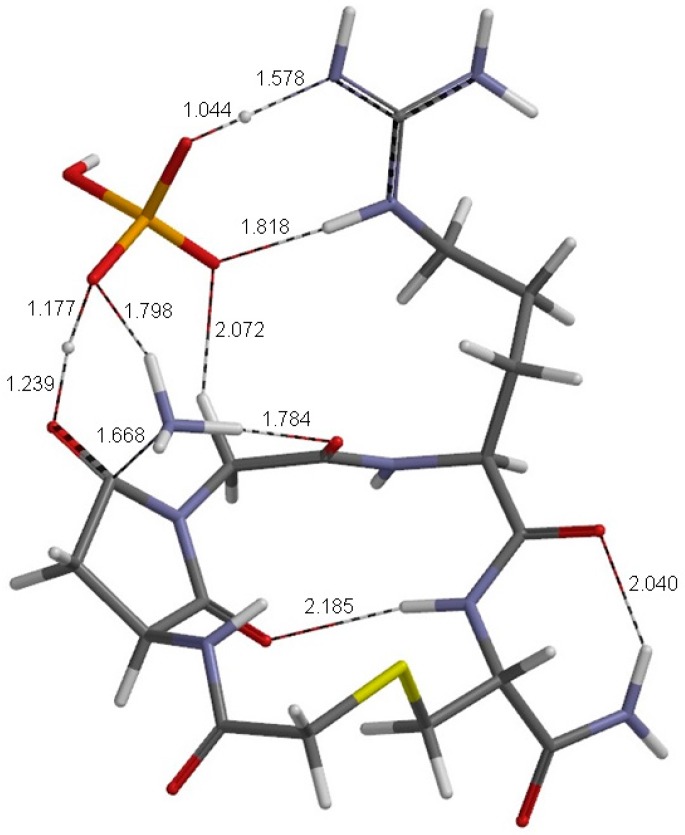
The geometry of TS2, the transition state of the second step (deammoniation) connecting TH2 (Figure 7) and PC (Figure 9). Selected interatomic distances are shown in Å. Grey: carbon; white: hydrogen; blue: nitrogen; red: oxygen; orange: phosphorus; yellow: sulfur.

**Figure 9:**
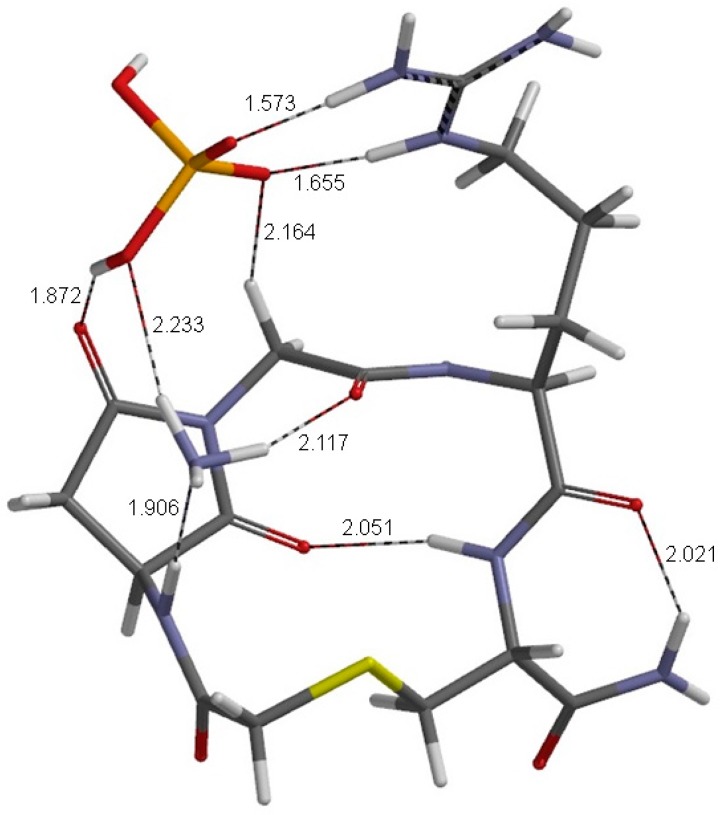
The geometry of PC (product complex) directly connected to TS2 (Figure 8). Selected interatomic distances are shown in Å. Grey: carbon; white: hydrogen; blue: nitrogen; red: oxygen; orange: phosphorus; yellow: sulfur.

Figure 2 shows the energy profile in water obtained from the present calculations, and Figure 3, Figure 4, Figure 5, Figure 6, Figure 7, Figure 8 and Figure 9 show optimized geometries. The geometry optimizations were performed in the gas phase by the density functional theory (DFT) with the B3LYP functional and the 6-31G(d) basis set, and the electronic energies were recalculated by using the 6-31+G(d,p) basis set at the optimized geometries. Furthermore, hydration Gibbs energies were calculated at the gas-phase optimized geometries by the SM8 (solvation model 8) continuum model [86,87] using the 6-31G(d) basis set. Relative energies were also corrected for the zero-point energy (ZPE). The Cartesian coordinates, total energies, ZPEs, and SM8 hydration Gibbs energies of the optimized geometries are provided in Tables S1–S7.

Figure 3 shows the optimized geometry of the reactant complex (RC) formed between CP15 (in the deprotonated form as shown in Figure 1) and an H_2_PO_4_^−^ ion. This geometry was obtained by an intrinsic reaction coordinate (IRC) calculation (followed by a full geometry optimization) from the transition state TS1 (transition state of the first step) described below; the geometry of TS1 was obtained after some trial and error. At pH 7.4, however, Arg side chains are in the protonated form, and HPO_4_^2−^ is the dominating phosphate species. We therefore attempted to find a complex between CP15 in the protonated form and an HPO_4_^2−^ ion near the RC geometry. However, in all attempts, a spontaneous proton transfer occurred from the protonated guanidino group to the HPO_4_^2−^ ion during geometry optimization. Since a reasonable activation barrier was obtained from RC (see below), we deduce that complex formation between CP15 and an HPO_4_^2−^ ion accompanies a simultaneous proton transfer from the former to the latter.

In RC (reactant complex), a nine-point interaction is observed between CP15 and the H_2_PO_4_^−^ ion comprising six hydrogen bonds and three CH–O interactions. These nine interactions are shown in Figure 4 as a–i, and the corresponding interatomic distances are shown in Table 1 (note that one of the two Arg β-protons is involved in a bifurcated CH–O interaction, the d and e interactions in Figure 4). It may be said that an HPO_4_^2−^ ion is efficiently recognized by the NGR motif of CP15 accompanied by a proton transfer. Interactions a and b are hydrogen bonding between the deprotonated guanidino group and the H_2_PO_4_^−^ ion. The main-chain NH group of Arg also forms a hydrogen bond to H_2_PO_4_^−^ (the interaction c). It is important that the neighboring amino acid residue of Asn on the C-terminal side is Gly. Otherwise, a pocket which can accommodate an H_2_PO_4_^−^ ion is not created because of the side chain. Indeed, the Gly α proton corresponding to the side chain of l-amino acid residues is involved in a CH–O interaction (the interaction i). Interactions h (2.235 Å) and g (1.893 Å) are important for the reaction because these are involved in the proton transfers which occur in the first step (see below). Interaction f involves the NH_2_ group of the Asn side chain. As a result, the Asn side chain is placed in the right position to undergo the cyclization (C–N bond formation); the distance between the carbon and nitrogen atoms to form a bond is 3.089 Å in RC.

Furthermore, we can see two hydrogen bonds involving the Gly C=O group inside the 15-memberd ring of RC (Figure 3), one to the Asn NH group (1.958 Å) and the other to the Cys NH group (1.623 Å); the latter is very short and is thought to have a large contribution to stabilizing the macrocycle conformation in RC. In Table 2, the heavy-atom dihedral angles *θ*_1_–*θ*_15_ defined for the 15-membered ring, as in Figure 1, are shown.

The transition state TS1 shown in Figure 5 connects RC and TH1 (the tetrahedral intermediate directly connected to TS1, Figure 6). The activation barrier of the first step (cyclization to form the tetrahedral intermediate TH1) was calculated to be 90.9 kJ·mol^−1^ after the ZPE and hydration Gibbs energy corrections. This value falls within the range of typical activation energies of Asn deamidation (80–100 kJ·mol^−1^) [1,4,5,11]. The relative energy of TH1 with respect to RC is 27.7 kJ·mol^−1^.

At TS1, the NH proton of Gly has already been abstracted by the O atom in H_2_PO_4_^−^ which had originally formed a hydrogen bond (the interaction h, 2.235 Å) to the NH proton in RC (the NH and OH distances in TS1 are 2.465 and 0.978 Å, respectively). Thus, the nucleophilicity of the Gly nitrogen is enhanced in the initial stage of the first step (note that amide nitrogens generally have low nucleophilicity [88]). The distance of the forming N–C bond in TS1 is 2.277 Å. On the other hand, another proton transfer is occurring from the H_2_PO_4_^−^ ion to the Asn side-chain oxygen; this proton transfer is not completed at TS1 as may be seen from the relevant interatomic distances shown in Figure 5 (1.412 and 1.070 Å). All of these changes take place in a single step, leading to the formation of a five-membered ring tetrahedral intermediate TH1 (Figure 6) bearing an NH_2_ and an OH group on the same carbon atom (a *gem*-hydroxylamine species). It should be noted that the two hydrogen bonds inside the 15-membered ring were maintained through the first step. In particular, the hydrogen bond involving the Cys NH group remained very short (1.588 and 1.595 Å in TS1 and TH1, respectively). Among the dihedral angles *θ*_1_–*θ*_15_, *θ*_1_ and *θ*_7_ show the largest changes (by about 26° and 25°, respectively) in the first step (Table 2); on the other hand, *θ*_2_ and *θ*_11_ remained almost unchanged. The interactions a–f and i (Figure 4) were also preserved in the first step as may be seen from Table 1. Note that the proton transfers have occurred along the hydrogen bonds corresponding to the interactions g and h.

The NH_2_ group on the five-membered ring of TH1 is expected to be easily protonated at neutral or physiological pH (see our recent paper [13]). Therefore, the second step (NH_3_ release) was calculated for the NH_2_-protonated form of the tetrahedral intermediate (TH2). Figure 7 shows the optimized geometry for TH2. The protonation induced a relatively large geometrical change and a considerable energy lowering. As to the 15-membered ring conformation, *θ*_5_ and *θ*_14_ changed by about 88°, and *θ*_1_, *θ*_7_, and *θ*_13_ changed by more than 50°. See Figure 7 for the hydrogen bonding and CH–O interaction scheme in TH2. The two hydrogen bonds inside the 15-membered ring of TH1 have disappeared; instead, a new transannular hydrogen bond (2.186 Å) has been formed between the C=O group of the five-membered ring and the Cys NH group. The protonated NH_2_ group is stabilized by two hydrogen bonds (1.456 and 1.840 Å).

It should be noted that the energy of TH2 can not be directly compared to that of TH1 because of a different stoichiometry. In order to compare the energies of TH1 and TH2, we have added the recommended value of −265.9 kcal·mol^−1^ (1 cal = 4.184 J) for the hydration Gibbs energy of proton [89] to the energy of TH1 (note that the electronic energy of a bare proton is zero). This strategy is the same as the one we have employed in a recent paper [13]. The energy diagram shown in Figure 2 was obtained by adding this value to the energies of RC, TS1, and TH1. The protonation of TH1 at the NH_2_ nitrogen resulted in stabilization as large as 126.8 kJ·mol^−1^. This large value can be interpreted as follows. The experimental Gibbs energies of protonation for primary alkylamines (RNH_2_) are about −60 kJ·mol^−1^ [90]. In the case of TH1/TH2, the protonated amino group is stabilized by two hydrogen bonds, one to the Gly oxygen (1.840 Å) and the other to the H_2_PO_4_^−^ ion (1.456 Å); the latter is very short and, therefore, is thought to have a large stabilizing effect. Also, the neighboring OH group may have an additional stabilization effect on TH2.

From TH2, the departure of an NH_3_ molecule (C–N bond cleavage) occurs via TS2 (the transition state of the second step) shown in Figure 8. At TS2, the distance of the cleaving C–N bond is 1.668 Å (the corresponding distance in TH2 was 1.543 Å). A double proton transfer also occurs in this second step from the OH group on the five-membered ring to the deprotonated guanidino group mediated by the H_2_PO_4_^−^ ion, leading to the succinimide product with the protonated guanidino group. This double proton transfer is asynchronous as may be seen from the relevant interatomic distances shown in Figure 8. Whereas the proton transfer from the OH group to H_2_PO_4_^−^ occurs in concert with the C–N bond cleavage, the transfer from H_2_PO_4_^−^ to the guanidino group occurs in the latest stage of the second step. The local activation barrier for the second step is only about 21 kJ·mol^−1^.

Figure 9 shows the resultant product complex (PC), which comprises the succinimide product (in which the guanidino group of Arg is protonated), an H_2_PO_4_^−^ ion, and an NH_3_ molecule. In PC, the NH_3_ molecule forms three hydrogen bonds, two to the product succinimide molecule and one to the H_2_PO_4_^−^ ion. The H_2_PO_4_^−^ ion forms a total of five hydrogen bonds. The transannular hydrogen bond in the 15-membered ring was preserved through the second step. The relative energy of PC with respect to TH2 is −73.8 kJ·mol^−1^.

## 3. Computational Methods

All calculations in this work were performed by DFT with the B3LYP functional using Spartan ’14 [91]. Geometry optimizations and vibrational frequency calculations were performed in the gas phase using the 6-31G(d) basis set, and the gas-phase electronic energies were recalculated using the 6-31+G(d,p) basis set. Moreover, hydration Gibbs energies were calculated at the gas-phase optimized geometries by the SM8 continuum model [86,87]; the 6-31G(d) basis set was used for these calculations because it produces stable partial atomic charges and is recommended by the developers of the SM8 model [92]. By the vibrational frequency calculations, all the geometries reported in this paper were confirmed to be an energy minimum (with no imaginary frequency) or a transition state (with a single imaginary frequency), and their relative energies were corrected for the ZPE. Moreover, IRC calculations were performed from the located transition states followed by full geometry optimizations in order to confirm the energy minima connected by each transition state.

## 4. Conclusions

By the B3LYP DFT method, we have computationally revealed a phosphate-catalyzed mechanism for the succinimide formation from an NGR-containing cyclic peptide, c[CH_2_CO-NGRC]-NH_2_, which we named CP15. From the reactant complex, in which an H_2_PO_4_^−^ ion is bound with CP15 (deprotonated form) by a nine-point interaction involving all of the Asn, Gly, and Arg residues, an intramolecular cyclization occurs with an H_2_PO_4_^−^-mediated double proton transfer to form a five-membered ring tetrahedral intermediate having an OH and an NH_2_ group on the same carbon atom. The calculated activation barrier for this step (90.9 kJ·mol^−1^) fell within the range of typical activation energies for nonenzymatic Asn deamidation. The tetrahedral intermediate, once protonated at the NH_2_ group, was shown to easily undergo NH_3_ elimination leading to the succinimide formation. Thus, this study has revealed possible catalytic roles of the phosphate buffer and the Arg guanidino group on the succinimide formation from the NGR motif. Further studies on the NGR deamidation are now in progress.

## Figures and Tables

**Table 1:** Interatomic distances (Å) in RC (Figure 3), TS1 (transition state of the first step) (Figure 5), and TH1 (tetrahedral intermediate directly connected to TS1) (Figure 6) corresponding to the interactions a–i shown in Figure 4.

Geometry	a	b	c	d	e	f	g	h	i
RC	1.824	1.789	1.713	2.361	2.421	2.196	1.893	2.235	2.306
TS1	1.745	1.828	1.805	2.482	2.584	2.135	1.070	0.978	2.531
TH1	1.747	1.840	1.750	2.509	2.738	2.104	1.001	0.977	2.355

**Table 2:** The heavy-atom dihedral angles *θ*_1_–*θ*_15_ (°) defined for the 15-membered ring as in Figure 1.

Geometry	*θ*_1_	*θ*_2_	*θ*_3_	*θ*_4_	*θ*_5_	*θ*_6_	*θ*_7_	*θ*_8_	*θ*_9_	*θ*_10_	*θ*_11_	*θ*_12_	*θ*_13_	*θ*_14_	*θ*_15_
RC	−146	−91	139	−78	−121	165	40	−33	−157	155	−63	159	−131	44	167
TS1	−165	−89	145	−91	−120	168	20	−18	−165	160	−64	158	−132	60	174
TH1	−172	−91	149	−85	−133	169	15	−14	−165	159	−63	165	−127	53	−179
TH2	−117	−115	166	−59	139	180	69	6	177	151	−82	175	−67	−35	162
TS2	−116	−112	169	−60	138	−180	68	7	177	151	−83	177	−68	−35	160
PC	−90	−108	170	−49	132	179	70	1	−179	152	−70	171	−57	−76	159

## Supplementary Materials

Supplementary materials can be found at www.mdpi.com/1422-0067/18/2/429/s1.

## Abbreviations

CP	Cyclic peptide
DFT	Density functional theory
IRC	Intrinsic reaction coordinate
*iso*DGR	*iso*Asp-Gly-Arg
NGR	Asn-Gly-Arg
PC	Product complex
RC	Reactant complex
TH	Tetrahedral intermediate
TS	Transition state
ZPE	Zero-point energy
