# Supplementary material for: Succinimide Formation from an NGR-Containing Cyclic Peptide: Computational Evidence for Catalytic Roles of Phosphate Buffer and the Arginine Side Chain
Source: Int J Mol Sci. 2017 Feb 16;18(2):429. doi: 10.3390/ijms18020429 (PMC5343963; doi:10.3390/ijms18020429)
Supplement: Supplementary file 1 [file ijms-18-00429-s001.pdf]

Supplementary Materials: Succinimide Formation from an NGR-Containing Cyclic Peptide: Computational Evidence for Catalytic Roles of Phosphate Buffer and the Arginine Side Chain

Ryota Kirikoshi, Noriyoshi Manabe and Ohgi Takahashi

Table S1. Total energies (au), zero-point energies (kJ mol^{-1}), and SM8 hydration Gibbs energies (kJ·mol^{-1}) of the B3LYP/6-31G(d) optimized geometries.

Geometry	Total Energy (6-31+G(d,p))	Zero-Point Energy	SM8 Hydration Gibbs Energy
RC	−2651.4960626	1446.1426	−285.0577935
TS1	−2651.4624276	1439.8544	−276.1713111
TH1	−2651.4879063	1448.9169	−281.5687406
TH2	−2651.9987504	1473.1895	−203.9645850
TS2	−2651.9920277	1463.6345	−190.9690752
PC	−2652.0266361	1467.7496	−199.1116750

Table S2. Cartesian coordinates (Å) of RC (the reactant complex).

Atom	*x*	*y*	*z*
6	−1.268893781	−3.222103538	1.442729806
8	−2.131822831	−3.293228303	2.310197985
7	−0.056452252	−2.624472168	1.636197682
6	0.579426916	−3.198945470	−1.331058007
7	0.288062400	−1.931533895	−1.732074937
1	0.768811222	−2.787650319	1.062549639
6	0.076052687	−1.502236579	2.554125476
15	3.455716789	−1.133937116	0.941039685
8	5.066983731	−1.191700508	0.881048893
8	2.878999716	−2.331752831	1.640277623
8	3.036945366	0.269515914	1.417355227
8	3.004602245	−1.199389325	−0.661544022
6	−0.464332422	−0.258858900	1.816355296
8	−1.647904505	−0.301705554	1.404428957
7	0.385905850	0.763439118	1.655931644
6	0.349606666	1.967161651	0.787600459
6	0.975558291	1.629592242	−0.606159930
6	1.688611300	2.795190384	−1.310206949
6	2.949361453	3.305496149	−0.574904169
6	−0.937884435	2.769356426	0.525761261
8	−0.809565358	3.973546664	0.235275103
7	−2.111045945	2.118583789	0.480112724
6	−3.305788295	2.612855059	−0.197777274
7	3.954323429	2.300404419	−0.283129149
6	4.998863835	1.945017035	−1.078170468
7	5.824442286	1.017602154	−0.691738418
7	5.164228514	2.697836449	−2.258884657
8	1.738134121	−3.619308309	−1.265937041
1	2.867793133	−2.146267269	−0.896155074
6	−0.593253888	−4.117453744	−0.998706950
6	−1.666758490	−3.619560648	−0.003335473
7	−2.382129453	−2.423828464	−0.498872214
6	−3.428014142	−2.532826771	−1.362833825

Table S2. *Cont.*

Atom	x	y	z
8	−3.728745120	−3.573044180	−1.940618703
6	−4.278147981	−1.264701389	−1.567754584
16	−3.338908329	0.310873975	−1.786402094
6	−4.195107589	1.425800754	−0.586864720
6	−4.120724522	3.599084918	0.685892329
8	−5.270904309	3.369317374	1.042040125
7	−3.438481134	4.733899353	0.983537571
1	5.406923483	−0.377485755	0.407163711
1	6.508534678	0.804726506	−1.416224328
1	5.826898480	2.266000504	−2.891684472
1	4.298575119	2.905200501	−2.745501503
1	3.725011877	1.609080292	0.451367117
1	1.973125998	2.444953098	−2.313743594
1	3.407659455	4.119576308	−1.148786380
1	2.648722343	3.755060789	0.379355287
1	1.003306333	3.640092042	−1.446927585
1	1.695768922	0.825459771	−0.444153312
1	0.179742991	1.240289161	−1.255058772
1	1.006641666	2.674151011	1.299192373
1	1.393025797	0.527570237	1.821884348
1	−2.985085976	3.166073471	−1.091760742
1	−2.094176853	1.126310033	0.776418925
1	−2.439840293	4.784303714	0.769169299
1	−3.850615188	5.361092167	1.660347400
1	−5.125354345	1.801997972	−1.015385489
1	−4.448098538	0.855716206	0.312071409
1	−0.647445965	−1.578409930	−1.572889619
1	1.059459769	−1.271041510	−1.678950433
1	−4.907114877	−1.450507066	−2.439683429
1	−4.930851124	−1.146591541	−0.694909019
1	−2.296931546	−1.577456304	0.069079897
1	−2.403111163	−4.422490472	0.091489636
1	−1.127581367	−4.356899485	−1.926559502
1	−0.153625887	−5.045823144	−0.623223455
1	1.127262510	−1.415150947	2.827914134
1	−0.547863126	−1.675535209	3.434523192

Table S3. Cartesian coordinates (Å) of TS1 (the transition state of the first step).

Atom	x	y	z
6	1.572151693	3.183039507	1.440736203
8	2.212147298	3.283008916	2.498549275
7	0.361587955	2.647795777	1.207701985
6	0.056922980	2.966414762	−1.026189468
7	0.193888397	1.718099715	−1.534974721
1	−2.046470886	3.170092649	1.142323311
6	−0.024465190	1.624740143	2.170528044
1	−1.101002016	1.603743289	2.354463207
1	0.494510403	1.784333314	3.126380495
15	−3.394288356	1.591155985	0.471589213
8	−4.961686127	1.847445047	0.301192038
8	−2.981149638	2.929517734	1.299275336
8	−3.146703233	0.321318604	1.284360497
8	−2.650831297	1.622223545	−0.885717933
1	−0.648262183	1.149506188	−1.453846874

Table S3. *Cont.*

Atom	*x*	*y*	*z*
1	−4.060443591	−1.108697819	0.605524195
6	0.457092970	0.274580520	1.612167234
8	1.678657136	0.125598640	1.377103350
7	−0.502040584	−0.645504179	1.389104418
1	−1.461934692	−0.280482234	1.520664139
6	−0.578787642	−1.952231420	0.690456694
6	−1.382965258	−1.768475271	−0.642857604
6	−2.331060955	−2.926773340	−0.993731629
6	−3.534887805	−3.065549717	−0.036239057
6	0.652902836	−2.819180630	0.361632130
8	0.421723612	−3.994370643	0.013509960
7	1.874994392	−2.276013056	0.374005902
1	1.950165001	−1.302121028	0.736766888
6	3.063597101	−2.892511405	−0.210805317
1	−1.169524110	−2.588208614	1.358107271
1	2.768176784	−3.380275632	−1.149669772
1	−0.670810307	−1.620561799	−1.464634340
1	−1.962727581	−0.846869358	−0.557446209
1	−2.703473095	−2.758377207	−2.015359604
1	−1.776036420	−3.870714462	−1.002625076
1	−4.130797184	−3.945523330	−0.307738193
1	−3.173701813	−3.263606513	0.979500274
7	−4.404718140	−1.904830980	0.048452128
6	−5.418450741	−1.601919963	−0.805193505
7	−6.054406992	−0.472180655	−0.687728402
7	−5.777387407	−2.601923738	−1.724847368
1	−5.422109539	0.993009455	0.017933298
1	−6.769835010	−0.365397339	−1.404204151
1	−6.355573388	−2.246441677	−2.475934214
1	−5.001964631	−3.139104627	−2.094706890
8	−1.111779025	3.532230769	−1.085029298
1	−1.874389984	2.781872804	−1.098745990
6	1.240285886	3.901647400	−0.966938421
6	2.270373333	3.520399064	0.100350377
1	1.714753864	3.934081426	−1.954586598
1	0.842339904	4.895351062	−0.744679264
1	3.002539972	4.324543688	0.216461649
7	2.985757423	2.278287544	−0.243873887
1	2.794363984	1.474707660	0.355234863
6	3.961854696	2.211906412	−1.179928531
8	4.297125605	3.154224574	−1.895407660
6	4.684570578	0.855292981	−1.287768978
1	5.457809557	0.968952097	−2.048698523
1	5.162635414	0.620330653	−0.330774404
16	3.588788714	−0.573998813	−1.721506233
6	4.124543199	−1.815467346	−0.461279701
1	4.330851709	−1.295894359	0.478681381
1	5.049337639	−2.298119080	−0.781055946
6	3.675303358	−3.989512176	0.710232143
8	4.788848222	−3.882176151	1.210494360
7	2.871573911	−5.073691371	0.865022995
1	3.146657737	−5.752629842	1.561822334
1	1.895225050	−5.007679168	0.567512200
1	1.063945977	1.245982220	−1.314112356

Table S4. Cartesian coordinates (Å) of TH1 (the first tetrahedral intermediate).

Atom	*x*	*y*	*z*
6	2.056717384	3.060826966	1.372567148
8	2.519587670	3.047226419	2.503085118
7	0.780846655	2.716530948	1.001900389
6	0.469232946	2.958363582	−0.460863386
7	0.327894759	1.778248064	−1.279444388
1	−2.287402038	3.620035809	0.837988776
6	0.036060221	1.790572994	1.832966133
15	−3.410601841	1.832096261	0.348655631
8	−4.979826091	2.081353646	0.137958661
8	−3.019006809	3.134516390	1.266740485
8	−3.198602829	0.557956721	1.181374711
8	−2.601887388	1.908901980	−0.945254696
6	0.407437300	0.353129519	1.417918862
8	1.625398588	0.096283280	1.288901624
7	−0.617167662	−0.488956202	1.234171909
6	−0.769294050	−1.850553618	0.663095521
6	−1.580375263	−1.722719063	−0.673373387
6	−2.586297393	−2.854290089	−0.932015859
6	−3.774956683	−2.872442110	0.052594388
6	0.408885838	−2.810615796	0.389299258
8	0.102474190	−3.982777932	0.096820680
7	1.667800880	−2.356662721	0.394531370
6	2.817741001	−3.077733191	−0.147817352
7	−4.591241957	−1.670846962	0.070150823
6	−5.592856299	−1.383657864	−0.804807676
7	−6.179015545	−0.223832600	−0.764116046
7	−5.993530082	−2.428447731	−1.656364944
8	−0.703836293	3.720631184	−0.564240818
1	−1.422663663	3.130752800	−0.934915835
6	1.712362091	3.782695472	−0.878558911
6	2.828015973	3.343472101	0.074013493
7	3.417850925	2.042372672	−0.278296615
6	4.319293866	1.873191307	−1.275131244
8	4.784833355	2.790963668	−1.944395170
6	4.805770582	0.425072849	−1.496374123
16	3.476241801	−0.842764028	−1.715301974
6	3.952068178	−2.086825319	−0.435009309
6	3.345839742	−4.171351756	0.827086621
8	4.461820300	−4.118613796	1.330711596
7	2.467206276	−5.187690897	1.024968770
1	−6.890312928	−0.131274179	−1.486517243
1	−6.553309503	−2.098145022	−2.432554052
1	−5.239079529	−3.018375723	−1.986953531
1	−4.206888905	−0.854934324	0.572198187
1	−4.414251690	−3.741083829	−0.148405510
1	−2.076277938	−3.822120375	−0.890935036
1	−3.403866344	−3.017377896	1.073690180
1	−2.969517559	−2.731513389	−1.956009919
1	−5.454765155	1.233739607	−0.129854212
1	−2.114621190	−0.769405111	−0.650599047
1	−0.871446609	−1.662386025	−1.508164150
1	−1.384744338	−2.392452748	1.388759171
1	−1.561776414	−0.048124546	1.311949571
1	−1.035533278	1.982709442	1.801314966

Table S4. *Cont.*

Atom	*x*	*y*	*z*
1	−0.586783025	1.343163363	−1.156432200
1	1.082027790	1.112560288	−1.143421605
1	0.386457187	1.937762368	2.859500750
1	1.488008775	4.842445046	−0.718636147
1	1.945699931	3.621735413	−1.931292229
1	3.622637779	4.080720254	0.202553518
1	5.439173246	0.443340072	−2.384154625
1	5.417150888	0.117897292	−0.641139817
1	3.056482787	1.231273638	0.218578577
1	4.830724466	−2.644904888	−0.763072837
1	4.214862909	−1.569480308	0.491995651
1	2.497811155	−3.583238723	−1.068813594
1	2.692280556	−5.855474871	1.749819894
1	1.500209395	−5.070674182	0.713667867
1	1.812852142	−1.383739085	0.725184128

Table S5. Cartesian coordinates (Å) of TH2 (the second tetrahedral intermediate).

Atom	*x*	*y*	*z*
6	1.554629130	−1.936943412	−1.187212148
8	2.110189441	−0.950436485	−1.660119256
7	0.200608084	−2.112294637	−1.151076111
6	−0.236619777	−3.262792862	−0.348454773
7	−0.809528695	−2.689607054	0.965048376
6	−0.648136615	−0.996473690	−1.535352347
15	−4.186568872	−1.764905862	−0.110134452
8	−4.828112777	−1.325949061	1.272371448
8	−5.358759989	−2.468058726	−1.005577344
8	−3.609542900	−0.592822716	−0.887792618
8	−3.212443069	−2.923331412	0.211962813
6	−0.339775158	0.190639334	−0.612036848
8	−0.134608275	0.021186053	0.596954320
7	−0.288458225	1.404444513	−1.202582730
6	−0.016052482	2.674192598	−0.519566098
6	−1.041724197	3.051233544	0.577674007
6	−2.261992940	3.840758257	0.076527759
6	−3.336453031	3.039376164	−0.684509429
6	1.435404305	2.835732596	0.002385735
8	1.713754831	3.870320458	0.619128827
7	2.359511967	1.901302094	−0.306005913
6	3.757374176	2.060465190	0.114972182
7	−3.982846859	2.010118268	0.115085876
6	−5.137176490	2.186692472	0.817831273
7	−5.789216325	1.149160085	1.257700185
7	−5.579258608	3.498873844	0.977427797
8	−1.179089958	−4.057844117	−0.932172460
1	−2.100173620	−3.699328478	−0.754248943
6	1.074587470	−4.048743671	−0.131864961
6	2.232012110	−3.081482249	−0.427250359
7	2.887647390	−2.504517760	0.736508035
6	4.255783563	−2.572532134	0.862209606
8	4.951044153	−3.367100023	0.250133679
6	4.863189422	−1.591335437	1.873649186
16	4.032826855	0.039510217	1.995017849

Table S5. *Cont.*

Atom	*x*	*y*	*z*
6	4.444753210	0.711064821	0.326715655
6	4.549806435	2.911269620	−0.920007340
8	5.452152059	2.447840383	−1.601874148
7	4.139765999	4.205197558	−0.971970751
1	−4.084223564	3.733649026	−1.091618829
1	−1.927179177	4.671258085	−0.562349451
1	−2.888146398	2.532725155	−1.545625182
1	−2.728433629	4.311538199	0.953845422
1	−5.225394736	−0.375092464	1.257129918
1	−1.359192546	2.147274793	1.104331540
1	−0.503729664	3.674940283	1.295663936
1	1.057199389	−4.864517982	−0.858579519
1	1.138859893	−4.488341110	0.866510590
1	4.809477901	−2.026384720	2.878108720
1	5.918706599	−1.474908599	1.616933080
1	−1.921542643	−2.670470848	0.835363440
1	−0.475008506	−1.712789341	1.108675143
1	−0.579979221	−3.283031571	1.763525632
1	2.470798532	−1.670036569	1.144657409
1	3.018131876	−3.547077382	−1.026802030
1	−1.712741061	−1.236967054	−1.417433550
1	−0.465792208	−0.750586754	−2.585619939
1	−0.395532040	1.429472316	−2.207028437
1	−0.074283814	3.433297778	−1.310104273
1	−3.811327914	1.034721581	−0.170843163
1	−6.601674609	1.399084732	1.815614562
1	−4.864257368	4.212701136	1.032072699
1	−6.283315919	3.614379781	1.694462726
1	−5.705507904	−1.810154117	−1.628569931
1	2.116605099	1.082981569	−0.857369575
1	3.729392540	2.631102140	1.050049112
1	3.373957566	4.532137700	−0.390690651
1	4.579060676	4.821420038	−1.641247596
1	4.141728896	0.002978765	−0.450569703
1	5.524924961	0.849548301	0.254156021

Table S6. Cartesian coordinates (Å) of TS2 (the transition state of the second step).

Atom	*x*	*y*	*z*
6	1.490995052	−1.909044499	−1.273409207
8	2.136114179	−0.952336320	−1.700402703
7	0.134611667	−1.979478998	−1.251765535
6	−0.411734772	−3.136688682	−0.502464793
7	−0.718492932	−2.508228966	1.011493420
1	−5.712047952	−1.802729789	−1.621619172
6	−0.647884246	−0.830180536	−1.653797370
15	−4.262927456	−1.738747678	−0.031858664
8	−4.961836825	−1.278554606	1.302710479
8	−5.380011259	−2.450795869	−0.980292832
8	−3.596637096	−0.609887063	−0.791379487
8	−3.336133254	−2.929799295	0.393405005
6	−0.313455539	0.343831891	−0.728608001
8	−0.171423020	0.183112290	0.490574904
7	−0.174317991	1.553060832	−1.317607881

Table S6. *Cont.*

Atom	*x*	*y*	*z*
6	0.147484734	2.795742333	−0.607968650
6	−0.900107994	3.223409931	0.446532475
6	−2.149242009	3.921521019	−0.116363935
6	−3.224722019	3.021870788	−0.758183533
6	1.578984464	2.850448217	−0.010219878
8	1.893730480	3.851494817	0.643775842
7	2.452399892	1.859985463	−0.291301728
6	3.832548448	1.906977854	0.210330553
7	−3.817254395	2.052737437	0.156589387
6	−4.957236737	2.270323678	0.872470552
7	−5.610227286	1.260758485	1.373426285
7	−5.384096722	3.589801605	0.982401356
8	−1.506964478	−3.664168982	−0.967448667
1	−2.545559951	−3.320137834	−0.386592803
6	0.831080615	−4.048601012	−0.381312305
6	2.059317388	−3.140880452	−0.555914985
7	2.675518053	−2.660522371	0.678778168
6	4.034077189	−2.794272268	0.863657542
8	4.720652169	−3.609458630	0.270468198
6	4.639685498	−1.855661171	1.917690465
16	3.879839595	−0.188986888	2.034736935
6	4.413210730	0.505580614	0.409474229
6	4.741369366	2.730009331	−0.749820926
8	5.653989214	2.225748692	−1.387614726
7	4.419324661	4.048875143	−0.789147077
1	−6.403115547	1.540883779	1.944574889
1	−6.085958025	3.746040289	1.693755387
1	−4.662778291	4.298986560	0.994753769
1	−3.671025244	1.063506228	−0.084124829
1	−4.005416285	3.656483981	−1.198789856
1	−1.851077357	4.682610288	−0.852981495
1	−2.788580817	2.447427626	−1.580610526
1	−2.606925484	4.481058766	0.712210616
1	−5.218030613	−0.266874104	1.316317099
1	−1.185133745	2.355960538	1.047549035
1	−0.382185534	3.923317395	1.106741932
1	0.170791679	3.568416430	−1.387074901
1	−0.197760231	1.581170237	−2.327474774
1	−1.718102882	−1.050878235	−1.547320892
1	−1.744720212	−2.608254655	1.166171641
1	−0.483862767	−1.490467976	1.023634989
1	−0.438994628	−0.590990224	−2.701474330
1	0.755789118	−4.754068039	−1.213619407
1	0.848695844	−4.626457906	0.546043755
1	2.853386286	−3.613343289	−1.138760821
1	4.524446009	−2.302634056	2.911679412
1	5.709304335	−1.781970485	1.707850924
1	2.304566740	−1.795721026	1.068946486
1	5.502547757	0.570472641	0.392268647
1	4.099984619	−0.155377097	−0.404225736
1	3.792664800	2.449653437	1.161608620
1	4.941506065	4.652088525	−1.408885360
1	3.643489321	4.411164754	−0.242906943
1	2.184400582	1.068785388	−0.870806277
1	−0.204135797	−3.001199644	1.742263644

Table S7. Cartesian coordinates (Å) of PC (the product complex).

Atom	*x*	*y*	*z*
6	1.557643548	−1.898092818	−1.408578888
8	2.189525481	−0.871680266	−1.585560623
7	0.187132388	−1.996529095	−1.616255822
6	−0.305595060	−3.282660121	−1.409179510
7	−0.042330280	−2.262889244	2.027993922
6	−0.623277877	−0.834967568	−1.945704218
15	−4.034668810	−1.674594722	0.320633309
8	−4.552054022	−1.114196848	1.621407534
8	−5.175971827	−2.577917197	−0.441813545
8	−3.509797777	−0.686064071	−0.724515385
8	−2.924288675	−2.801053005	0.704442789
6	−0.324427384	0.317936220	−0.975442871
8	−0.337975783	0.168669708	0.237325949
7	−0.098910901	1.527686083	−1.573097765
6	0.178922270	2.770492048	−0.848747950
6	−0.938146955	3.203083023	0.129122824
6	−2.197456290	3.789102299	−0.529960319
6	−3.222037584	2.784283806	−1.092774018
6	1.554449706	2.861991933	−0.132390916
8	1.819428173	3.924186001	0.447327809
7	2.421766913	1.836855338	−0.215866817
6	3.724987653	1.890518939	0.464284598
7	−3.833486129	1.913846225	−0.089913616
6	−4.774787015	2.289730191	0.772244371
7	−5.248270563	1.432517633	1.673337682
7	−5.292350997	3.557372307	0.735508591
8	−1.471663229	−3.605537679	−1.543539775
1	−2.613502673	−3.262345332	−0.100602903
6	0.863257593	−4.179607215	−1.032436576
6	2.059315296	−3.234505767	−0.834679764
7	2.389101948	−3.016208620	0.561261369
6	3.662550649	−2.626526576	0.882709971
8	4.623431290	−2.764420198	0.133128674
6	3.807824331	−1.941776271	2.236480416
16	3.186672085	−0.206847780	2.177153446
6	4.232290716	0.496875192	0.833032146
6	4.768225850	2.618171985	−0.428981941
8	5.690717853	2.033427534	−0.976718092
7	4.550361981	3.958103090	−0.519847725
1	−4.001314579	3.322479647	−1.653119645
1	−1.912149687	4.467601186	−1.346524890
1	−2.740193483	2.111725657	−1.804676086
1	−2.695375974	4.421835459	0.217254668
1	−4.952033089	0.406596428	1.671374804
1	−1.192487188	2.364170293	0.782168925
1	−0.488206295	3.977801000	0.754583463
1	1.025013754	−4.898963528	−1.842855458
1	0.617736329	−4.745400083	−0.130347451
1	3.201731977	−2.426021841	3.008696277
1	4.859927146	−1.967406698	2.527026220
1	−0.972317512	−2.605403341	1.770340612
1	−0.051486159	−1.278562903	1.755313895
1	−0.008071405	−2.284157682	3.046867678
1	1.588374474	−2.765138044	1.178064328
1	2.971559290	−3.541796044	−1.348974854

Table S7. *Cont.*

Atom	*x*	*y*	*z*
1	−1.679652845	−1.095873770	−1.804681011
1	−0.448199028	−0.551003759	−2.989555577
1	0.089296478	1.524088784	−2.565972403
1	0.250265977	3.542576514	−1.625222300
1	−3.663257754	0.879671379	−0.210258471
1	−6.096316798	1.671330559	2.167510693
1	−4.809309155	4.257110759	0.193866932
1	−5.715252525	3.907647092	1.583429656
1	−5.295509983	−2.197373401	−1.325700114
1	2.175762093	0.977908271	−0.699678397
1	3.576885792	2.495751528	1.365669291
1	3.705657926	4.365374541	−0.127696259
1	5.129626227	4.490702386	−1.153777966
1	4.225547443	−0.160290506	−0.038817387
1	5.266180952	0.580967092	1.174633412
